# Influence of a Short Course of Ritonavir Used as Booster in Antiviral Therapies Against SARS-CoV-2 on the Exposure of Atorvastatin and Rosuvastatin

**DOI:** 10.1007/s10557-023-07538-w

**Published:** 2023-12-19

**Authors:** Evelyn Krohmer, Brit Silja Rohr, Felicitas Stoll, Katja S. Gümüs, Mariano Bergamino, Gerd Mikus, Max Sauter, Jürgen Burhenne, Johanna Weiss, Andreas D. Meid, David Czock, Antje Blank, Walter E. Haefeli

**Affiliations:** Department of Clinical Pharmacology and Pharmacoepidemiology, University of Heidelberg, Medical Faculty of Heidelberg, Im Neuenheimer Feld 410, 69120 Heidelberg, Germany

**Keywords:** Rosuvastatin, Atorvastatin, Nirmatrelvir-ritonavir, Drug-drug interaction, Healthy volunteers

## Abstract

**Purpose:**

Early antiviral treatment with nirmatrelvir/ritonavir is recommended for SARS-CoV-2-infected patients at high risk for severe courses. Such patients are usually chronically ill and susceptible to adverse drug interactions caused by ritonavir. We investigated the interactions of short-term low-dose ritonavir therapy with atorvastatin and rosuvastatin, two statins commonly used in this population.

**Method:**

We assessed exposure changes (area under the concentration–time curve (AUC_∞_) and maximum concentration (*C*_max_)) of a single dose of 10 mg atorvastatin and 10 mg rosuvastatin before and on the fifth day of ritonavir treatment (2 × 100 mg/day) in healthy volunteers and developed a semi-mechanistic pharmacokinetic model to estimate dose adjustment of atorvastatin during ritonavir treatment.

**Results:**

By the fifth day of ritonavir treatment, the AUC_∞_ of atorvastatin increased 4.76-fold and *C*_max_ 3.78-fold, and concurrently, the concentration of atorvastatin metabolites decreased to values below the lower limit of quantification. Pharmacokinetic modelling indicated that a stepwise reduction in atorvastatin dose during ritonavir treatment with a stepwise increase up to 4 days after ritonavir discontinuation can keep atorvastatin exposure within safe and effective margins. Rosuvastatin pharmacokinetics were only mildly modified; ritonavir significantly increased the *C*_max_ 1.94-fold, while AUC_∞_ was unchanged.

**Conclusion:**

Atorvastatin doses should likely be adjusted during nirmatrelvir/ritonavir treatment. For patients on a 20-mg dose, we recommend half of the original dose. In patients taking 40 mg or more, a quarter of the dose should be taken until 2 days after discontinuation of nirmatrelvir/ritonavir. Patients receiving rosuvastatin do not need to change their treatment regimen.

**Trial Registration:**

EudraCT number: 2021–006634-39.

DRKS00027838.

**Supplementary Information:**

The online version contains supplementary material available at 10.1007/s10557-023-07538-w.

## Introduction

Nirmatrelvir/ritonavir (Paxlovid®) was approved in January 2022 for the early treatment of SARS-CoV-2-infected patients at high risk of severe courses. Its component ritonavir is used as a boosting agent to reach and maintain therapeutic drug concentrations of nirmatrelvir throughout the dosing interval by inhibiting a variety of cytochrome P450 (CYP) isozymes including CYP3A4, which is the main CYP involved in nirmatrelvir metabolism. Older patients or patients with chronic conditions such as cardiovascular disease or diabetes have a higher risk of suffering a severe course of SARS-CoV-2 and often concurrently take additional medications [1]. Intake of nirmatrelvir/ritonavir within 3 days after being positive for SARS-CoV-2 significantly reduces the risk in this population [2]. Complicating matters further, patients with mild symptoms of SARS-CoV-2 infection are usually treated at home, where surveillance is limited and adverse effects from drug-drug interactions (DDIs) may go unnoticed. Recommendations for the management of DDIs between nirmatrelvir/ritonavir and other medicines [3] are based on assumptions derived from data available from long-term treatment with ritonavir as it is used in life-long antiviral therapy for patients infected with the human immunodeficiency virus [4]. However, previous DDI studies often used higher ritonavir doses or longer treatment periods, so their results may not adequately reflect the magnitude of the interaction observed with short-term treatment with small booster doses.

Ritonavir primarily and irreversibly inhibits CYP3A4 and to lesser extent CYP2D6, CYP2C19, CYP2C8, and CYP2C9 [5, 6]. It also inhibits hepatic uptake transporters such as several organic anion-transporting polypeptides (OATP) and efflux transporters such as breast cancer resistance protein (BCRP) and P-glycoprotein [7]. In addition, it induces CYP1A2, CYP2B6, CYP2C9, and CYP2C19 [8]. The net sum of the effect within the first 5 days remains unclear but is likely dominated by rapid inhibition [9], while treatment appears to be too short for induction of CYP3A albeit possibly not for all CYP isozymes [10].

Statins are widely used as cholesterol-lowering drugs and are one of the mainstays in the prevention and treatment of atherosclerotic vascular disease, as they reduce the risk of major cardiovascular and cerebrovascular events and cardiovascular mortality [11]. The different statins have very different metabolic pathways, which determine their risk profile for DDIs. Atorvastatin is mainly metabolized by CYP3A4 to form its two active metabolites 2-hydroxy atorvastatin and 4-hydroxy atorvastatin [12], while CYP-mediated metabolism of rosuvastatin to its active metabolite *N*-desmethyl rosuvastatin is small (10% of the dose) [13]. In addition to a reduction in CYP activity, functional genetic polymorphisms [14] and inhibition of hepatic uptake transporters such as several OATPs or the efflux transporter BCRP can also increase systemic exposure and the risk of adverse events [15]. Rhabdomyolysis due to statin therapy is a rare but serious adverse event [16], and the risk increases with exposure, which is why high statin doses [17] and impaired drug clearance increase its likelihood [18]. Even though rhabdomyolysis often evolves over a time course of months, there are case reports describing the onset of rhabdomyolysis within 1 week [19, 20]. Strong inhibition of statin clearance by ritonavir therefore likely poses a risk particularly in patients on high statin doses.

We therefore aimed to assess the DDI potential of a short-term ritonavir treatment course on the exposure of atorvastatin and rosuvastatin. In addition, a microdose of midazolam was administered to evaluate the contribution of CYP3A4 to the overall extent of the DDI.

## Material and Methods

### Clinical Trial

This was a single-center, open-label, two-arm, phase I DDI trial, recruiting eight healthy volunteers in each arm. The trial started after a positive vote of the responsible Ethics Committee of the Medical Faculty of Heidelberg University (ethical approval number: AFmo-956/2021) and the approval of the competent authority (Bundesinstitut für Arzneimittel- und Medizinprodukte, BfArM, EudraCT 2021–006634-39). All procedures were carried out according to the Good Clinical Practice (GCP) guideline, the pertinent version of the Declaration of Helsinki, and all legal requirements in Germany. The study was prospectively registered in the German Clinical Trials Register (DRKS-ID: DRKS00027838) and was conducted at the early clinical trial unit (KliPS) of the Department of Clinical Pharmacology and Pharmacoepidemiology, Heidelberg University Hospital, which is certified according to DIN EN ISO 9001:2015.

### Study Population

Before the start of any study-related procedures, all participants gave their written informed consent. Participants of the rosuvastatin part of the trial underwent post hoc genotyping if they had previously consented to a genotyping biobank study (ethical approval number: S-026/2004). In a screening phase, healthy volunteers aged 18–60 years were examined for relevant underlying diseases. For this purpose, medical history, physical examination, 12-lead electrocardiogram, routine laboratory parameters (including blood biochemistry, blood cell count, urinalysis, pregnancy test (females of childbearing potential), and screening for illicit drugs), and intake of prior medication were evaluated. Any intake of alcohol, medicines (except oral contraceptives and levothyroxine), and also vaccinations were prohibited 2 weeks prior to the first study drug intake and throughout the trial.

### Trial Design

We evaluated the effect of a 5-day short-term low-dose ritonavir treatment course (twice daily Norvir® 100 mg tablets, AbbVie Deutschland GmbH & Co. KG, Ludwigshafen, Germany) on the pharmacokinetics (PK) of a single dose of 10 mg atorvastatin (Atorvastatin-ratiopharm®, Ratiopharm GmbH, Ulm, Germany) or a single dose of 10 mg rosuvastatin (Rosuvastatin-ratiopharm®, Ratiopharm GmbH, Ulm, Germany). The PK of the statins were evaluated at baseline and during ritonavir on treatment day 5 (Online Resource 1). Midazolam (30 µg at baseline or 10 µg during ritonavir, diluted in tap water, Dormicum® V 5 mg/5 mL, CHEPLAPHARM Arzneimittel GmbH, Greifswald, Germany) was used as a marker substrate to assess CYP3A4 activity.

On trial days, participants arrived in a fasting state (> 6 h); food intake was not allowed until 4 h after midazolam administration. Blood samples were obtained via a peripheral venous catheter before and 15, 30, 45 min, and 1, 1.5, 2, 2.5, 3, 4, 5, 6, 8, 24, 25, 48, and 49 h after the administration of statins and 2, 2.5, 3, and 4 h after the midazolam administration according to a previously published limited sampling strategy [22, 23].

### Analytical Procedures

Blood samples for midazolam and atorvastatin were centrifuged at room temperature and for rosuvastatin at − 4 °C (2500 g, 10 min). The plasma was separated, and a NH_4_Ac buffer (5 M, pH 4.5) was added (25 µL) to the rosuvastatin samples (500 µL) to stabilize acid and lactone forms. All samples were stored at − 20 °C until analysis.

Midazolam plasma concentrations were quantified with ultra-performance liquid chromatography coupled to tandem mass spectrometry (UPLC-MS/MS) in the analytical laboratory of the department [24]. The lower limit of quantification (LLOQ) was 1 pg/mL. Interday accuracy (precision) varied from + 11.1 (1.59) to + 12.1% (2.59%).

Acid and lactone forms of atorvastatin, its hydroxylated metabolites, 2-hydroxy and 4-hydroxy atorvastatin, rosuvastatin, and its metabolite *N*-desmethyl rosuvastatin concentrations were quantified in plasma with UPLC-MS/MS methods developed and validated according to the ICH M10 guideline on bioanalytical method validation [25]. Both drugs and all metabolites fulfilled the ICH validation criteria. The LLOQ for atorvastatin and rosuvastatin (and all their metabolites) was 0.1 and 0.05 ng/mL, respectively. Interday accuracy (precision) varied from + 0.21 (2.30) to + 1.35% (4.69%) for atorvastatin and from − 12.2 (2.08) to − 7.99% (8.46%) for rosuvastatin.

### Genotyping

Genotyping of *SLCO1B1* polymorphisms was conducted for the participants of the rosuvastatin group with a LightCycler 480-based method with hybridization probes for the two single nucleotide polymorphisms c.521 T > C (V174A, rs4149056) and c.388A > G (N130D, rs2306283) [26]. The resulting genotypes are defined as SLCO1B1*1 (c.521 T, c.388A, formerly *1A) and *37 (c.521 T, c.388G, formerly *1B), *5 (c.521C, c.388A), and *15 (c.521C, c.388G). Carriers with SLCO1B1*1 or *37 alleles have normal OATP1B1 function (= wildtype), whereas SLCO1B1*5 and *15 are poor function alleles. Heterozygous carriers have one normal and one poor function allele and homozygous carriers have two poor function alleles [27].

### Pharmacokinetic Analysis

Standard PK measures and parameters, including area under the plasma concentration–time curve, extrapolated to infinity (AUC_∞_), maximum concentration (*C*_max_), time to reach *C*_max_ (*T*_max_), and half-life (*t*_1/2_) of atorvastatin, rosuvastatin, their relevant metabolites, and area under the plasma concentration–time curve from 2 to 4 h (AUC_2–4_) of midazolam (normalized to 1 µg for the two doses administered because of their linear PK) [28] were calculated with Phoenix WinNonlin Software 8.3 (Certara, Princeton, NJ, USA) with noncompartmental analysis (NCA). Atorvastatin and its metabolites are equipotent [29]; therefore, total HMG-CoA reductase inhibitory activity was calculated as the sum of their exposures (molar AUC_∞_). For rosuvastatin, more than 90% of HMG-CoA-reductase inhibitory activity is attributed to the parent, and *N*-desmethyl rosuvastatin is 50% less active. Therefore, the total HMG-CoA reductase inhibitory activity of rosuvastatin was not calculated [30].

### Modelling and Simulation

A semi-mechanistic PK model was developed to model atorvastatin PK as a function of ritonavir-modulated atorvastatin absorption and elimination. First, a two-compartment PK model with zero-order absorption characteristics was developed for atorvastatin, based on the measured concentrations without ritonavir. Then, systemic clearance and bioavailability of atorvastatin were tested for CYP-dependent modulation by ritonavir, using a turn-over model for CYP activity allowing for a time-dependent change in the amount of functional CYP enzyme in the gut and liver related to ritonavir intake [31] (Online Resource 2). With this structural model, parameters were estimated with the nlmixr2 R package (nlmixr2est version 2.1.3) [32] to derive a mixed-effects population PK model using the first order–conditional estimation with interaction (FOCEI) algorithm [33]. Initial estimates for fixed effects were informed by the NCA analysis and preliminary individual fits obtained with Phoenix WinNonlin 8.3, which also determined the apparent zero-order absorption time. Inter-individual variability (IIV) was allowed for relative bioavailability, apparent systemic clearance, and apparent central volume of distribution (Online Resource 3). Visual goodness-of-fit inspection, likelihood-based objective function values, standard errors of the parameter estimates, and biological plausibility of the parameter values were used to compare alternative models (Online Resource Fig. 4). Finally, steady-state atorvastatin concentrations without ritonavir and the impact of 5 days of ritonavir intake were predicted with the final model using the rxode2 R package (version 2.0.9) [34] for different atorvastatin dosing schemes.

### Statistics

Statistical analyses were performed with Prism 9.1.1 (GraphPad Software, La Jolla, CA, USA). According to the guideline for evaluation of bioequivalence [35], changes in exposure were evaluated by assessing the geometric mean ratio (GMR, paired *t* test on logarithmic transformed values) of AUC_∞_ and *C*_max_ during ritonavir divided by the corresponding values at baseline using the 90% confidence interval (CI). Changes for other PK parameters were assessed by calculating GMR with 90% CI. All other PK values are described by their geometric mean with a 95% CI unless indicated otherwise. A *p*-value < 0.05 was considered statistically significant.

## Results

### Population

Eight healthy White volunteers (7 females/1 male) with a median age of 25 years (range 20–58) and a mean (± standard deviation) body mass index (BMI) of 24.3 ± 2.46 kg/m^2^ were enrolled in the atorvastatin group, and eight healthy White volunteers (5 females/3 males) with a median age of 27 years (range 20–58) and a BMI of 24.1 ± 2.24 kg/m^2^ in the rosuvastatin group. One participant in each group took levothyroxine. Two participants in the atorvastatin and one in the rosuvastatin group were on oral contraceptives. All 16 participants completed the trial.

### Effect of Ritonavir on the Pharmacokinetics of Atorvastatin and Atorvastatin Metabolites

**Fig. 1:**
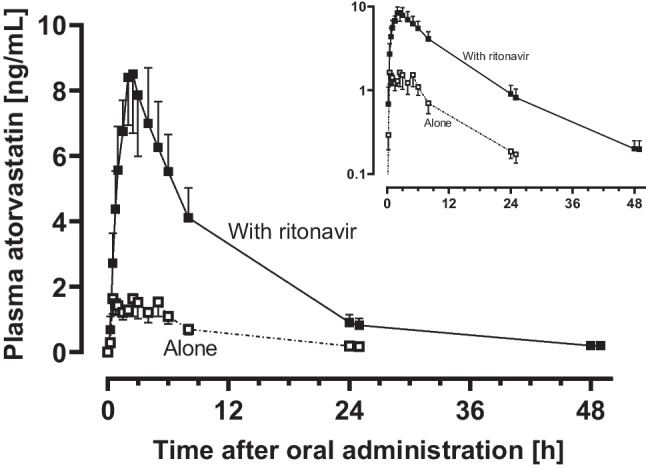
Mean (± SEM) plasma concentration–time profiles of atorvastatin before and on the fifth day of ritonavir administration (2 × 100 mg/day) in eight healthy volunteers

**Fig. 2:**
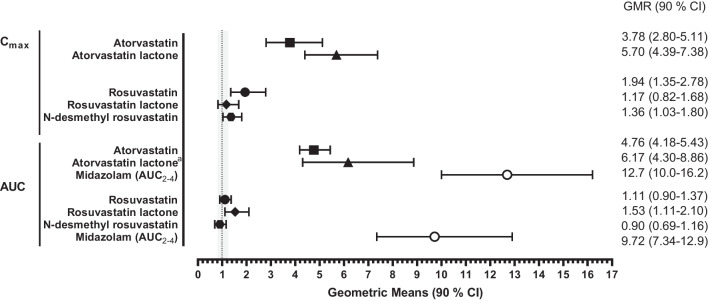
Impact of 5 days of ritonavir (2 × 100 mg/day) on the exposure of atorvastatin, rosuvastatin, their corresponding lactone forms, and metabolites and on midazolam in healthy volunteers. ^a^*n* = 7 (for one participant calculation of *t*_1/2_ was not possible). AUC, area under the concentration–time curve extrapolated to infinity (AUC_∞_) unless indicated otherwise; AUC_2–4_, area under the concentration–time curve from 2 to 4 h; *C*_max_, maximum plasma concentration; CI, confidence interval; GMR, geometric mean ratio (comparison with baseline). The grey area indicates the bioequivalence range, and the dotted line is the line of no change

The mean plasma concentration–time curves of atorvastatin increased substantially after 5 days of ritonavir treatment (Fig. 1) as did the AUC_∞_ and *C*_max_ of atorvastatin and its lactone form (both *p* < 0.0001) (Fig. 2 and Table 1). The 2-hydroxy atorvastatin metabolite was measurable at baseline and plasma concentrations decreased below the LLOQ on the fifth day of ritonavir treatment (Online Resource 5) while 4-hydroxy atorvastatin concentrations were below the LLOQ on both study days. The plasma ratio of atorvastatin and its lactone was 1.84 at baseline and 1.43 on the fifth day of ritonavir treatment; the corresponding plasma ratio of the metabolite was 1.21 at baseline and not quantifiable during ritonavir. Total geometric mean inhibitory HMG-CoA-activity (± 95% CI) increased from 76.5 h•mmol/L (58.2–101 h•mmol/L) at baseline to 152.0 h•mmol/L (102–225 h•mmol/L) during ritonavir treatment.

**Table 1:** Pharmacokinetic parameters of atorvastatin and 2-hydroxy atorvastatin before and on the fifth day of ritonavir administration (2 × 100 mg/day) to eight healthy volunteers

PK parameter	Baseline	Day 5 of ritonavir	Baseline	Day 5 of ritonavir
	Atorvastatin		Atorvastatin lactone	
AUC_∞_[h • ng/mL]	17.8 (13.0–24.4)	84.9 (57.2–126)	9.61^a^ (6.10–15.1)	59.3^a^ (34.0–104)
*C*_max_[ng/mL]	2.27 (1.42–3.60)	8.57 (5.26–14.0)	0.75 (0.54–1.05)	4.29 (2.85–6.45)
Median *t*_max_ (range)[h]	1.25 (0.50–5.00)	2.00 (1.50–8.00)	4.00 (1.00–6.00)	3.50 (2.50–8.00)
*t*_1/2_[h]	9.07 (7.20–11.4)	10.2 (6.91–15.2)	8.23^a^ (6.00–11.3)	9.91^a^ (7.98–12.3)
CL/*F*[mL/min]	9353 (6823–12,819)	1963 (1323–2913)	17,350^a^ (11,014–27,331)	2811^a^ (1610–4908)
*V*_z_/*F*[L]	7346 (4837–11,155)	1741 (996–3045)	12,357^a^ (8800–17,351)	2411^a^ (1482–3922)
	2-Hydroxy atorvastatin		2-Hydroxy atorvastatin lactone	
AUC_∞_[h • ng/mL]	25.4 (19.4–33.2)	n.d	25.9 (16.6–40.3)	n.d
*C*_max_[ng/mL]	1.43 (0.98–2.08)	n.d	1.44 (0.93–2.25)	n.d
Median *t*_max_ (range)[h]	5.00 (0.75–6.00)	n.d	5.00 (2.00–6.00)	n.d
*t*_1/2_[h]	6.35 (2.83–14.3)	n.d	12.0 (7.33–19.8)	n.d

Values are shown as geometric mean (95% confidence interval)

*AUC*_*∞*_ area under the plasma concentration–time curve extrapolated to infinity, *CL/F* apparent clearance after oral administration, *C*_*max*_ maximum concentration, *n.d.* not detectable, *t*_*max*_ time to reach *C*_max_, *t*_*1/2*_ terminal half-life, *V*_*z*_*/F* apparent volume of distribution

^a^*n* = 7 (for one participant calculation of *t*_1/2_ was not possible)

### Effect of Ritonavir on the Pharmacokinetics of Rosuvastatin and N-Desmethyl-Rosuvastatin

Five days of ritonavir did not significantly change the AUC_∞_ of rosuvastatin and *N*-desmethyl rosuvastatin but increased the AUC_∞_ of rosuvastatin lactone by 53% (Online Resource 6). Concurrently, *C*_max_ of rosuvastatin increased by 94% (Table 2 and Fig. 2).

**Table 2:** Pharmacokinetic parameters of rosuvastatin and *N*-desmethyl rosuvastatin at baseline and on the fifth administration day of ritonavir (2 × 100 mg/day) in eight healthy volunteers

PK parameter	Rosuvastatin		Rosuvastatin lactone		*N-*desmethyl rosuvastatin	
	Baseline	5 day of ritonavir	Baseline	5 day of ritonavir	Baseline	5 day of ritonavir
AUC_∞_[h • ng/mL]	38.9 (24.7–61.3)	43.2 (31.9–58.7)	13.7 (10.9–17.2)	20.9 (14.2–30.8)	4.28 (2.05–8.93)	3.35 (1.94–5.79)
AUCτ[h • ng/mL]	36.8^a^ (23.0–58.8)	40.5 (29.8–55.1)				
*C*_max_[ng/mL]	4.04 (2.64–6.18)	7.82 (5.56–11.0)	0.79 (0.62–1.02)	0.94 (0.57–1.53)	0.52 (0.28–0.98)	0.71 (0.42–1.20)
Median *t*_max_[h](range)	4.00 (0.50–5.00)	0.75 (0.50–1.00)	3.50 (1.50–8.00)	3.50 (1.00–6.00)	3.50 (1.50–5.00)	0.88 (0.75–4.00)
*t*_1/2_[h]	10.9^a^ (7.19–16.4)	17.0 (12.8–22.5)	14.4 (10.8–19.2)	18.4 (12.6–26.9)	3.11 (1.58–6.13)	2.54 (1.58–4.28)
CL/*F*[mL/min]	4286 (2720–6753)	3854 (2841–5228)	12,154 (9694–15,237)	7966 (5420–11,708)		
*V*_z_/*F*[L]	4026 (2120–7644)	5661 (3526–9089)	15,170 (10,067–22,859)	12,688 (6566–24,516)		

Values are shown as geometric mean (95% confidence interval)

*AUC*_*∞*_ area under the plasma concentration–time curve extrapolated to infinity, *CL/F* apparent clearance after oral administration, *C*_*max*_ maximum concentration, *t*_*max*_ time to reach *C*_max_, *t*_*1/2*_ terminal half-life, *V*_*z*_*/F* apparent volume of distribution

^a^Two profiles were below the LLOQ after 25 h

**Fig. 3:**
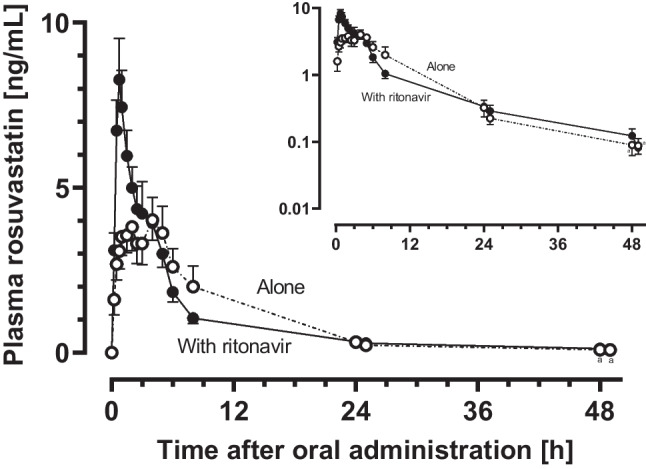
Mean (± SEM) plasma concentration–time profiles of rosuvastatin before and on the fifth day of ritonavir administration (2 × 100 mg/day) in eight healthy volunteers. ^a^*n* = 6 (two profiles were below the LLOQ after 25 h)

**Fig. 4:**
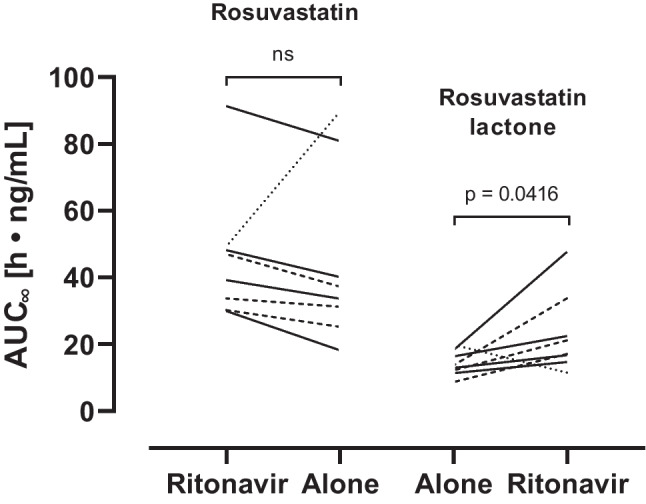
Impact of 5 days of ritonavir (2 × 100 mg/day) on the exposure of rosuvastatin after the administration of a single dose of 10 mg to eight healthy volunteers. The homozygous carrier of defective *SLCO1B1* alleles is indicated with a dotted line, heterozygous carriers with dashed lines, and solid lines indicate carriers who have only alleles coding for normal uptake transporter function. AUC_∞_, area under the concentration–time curve extrapolated to infinity

The mean plasma concentration–time curves of rosuvastatin are shown in Fig. 3 and individual exposure changes in Fig. 4.

### Relationship Between SLCO1B1 Genotypes and Rosuvastatin Pharmacokinetics

Plasma rosuvastatin concentrations increased during ritonavir in all but one participant. This latter participant (dotted line in Fig. 4) had a GMR of 0.45 and was a carrier of SLCO1B1*5/*15, i.e., two *SLCO1B1* alleles coding for poor OATP1B1 transport function. Three other participants (dashed lines in Fig. 4) were heterozygote but showed no significant change in rosuvastatin exposure and are comparable to wildtype carriers. When the participant with opposing results was excluded from the analysis, the GMR was 1.23 (1.11–1.36; *p* = 0.0074) for AUC_∞_ and 2.28 (1.84–2.83; *p* = 0.0192) for *C*_max_. There was no obvious relationship between rosuvastatin exposure and genetic OATP1B1 transporter function during baseline.

### CYP3A4 Inhibition by Ritonavir as Measured by Midazolam Exposure

Ritonavir profoundly increased midazolam exposure in all participants (Fig. 2). AUC_2–4_ was elevated 12.7-fold in the atorvastatin group and 9.72-fold in the rosuvastatin group (Online Resource 7). A regression analysis between individual atorvastatin AUC ratios and midazolam ratios however showed no statistically significant correlation (*r*^2^ = 0.02. *p* = 0.75) (Online Resource 8).

### Population Pharmacokinetic Modelling and Simulation

**Fig. 5:**
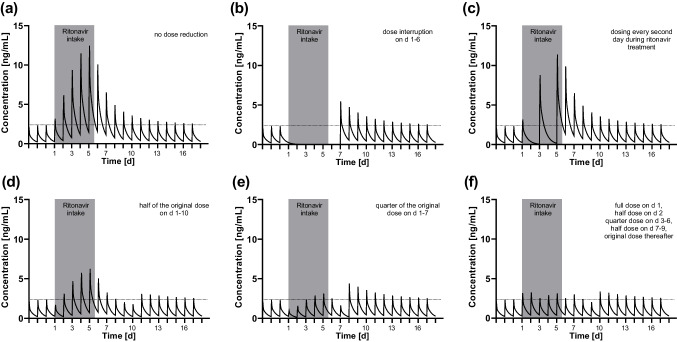
Predicted atorvastatin concentrations (population mean) during 5 days ritonavir treatment without dose adjustment (**a**), with treatment interruption (**b**), atorvastatin administration only every second day (**c**), dose reduction to half of the original dose on days 1–10 (**d**), dose reduction to quarter of the original dose on days 1–7 (**e**), and a mixed dosing schedule (**f**)

In population PK modelling of atorvastatin, a 2-compartment model performed best when absorption followed a zero-order process. Ritonavir modulated this absorption process with prolonged absorption time (factor 1.71) and increased relative bioavailability (factor 1/0.57 = 1.75), while no additional effect on elimination was detected (Online Resource Table S1). Using this model, the PK simulations of atorvastatin concentrations with different dosing schemes (Fig. 5) revealed that a daily dose reduction with a mixed dosing schedule (Fig. 5f) was most effective to keep atorvastatin exposure stable, while a simplified schedule appeared most practical (Fig. 5e). Keeping the original dose while prolonging its dosing interval (Fig. 5c) resulted in 4.8-fold higher *C*_max_ values. With temporary discontinuation for the duration of ritonavir treatment, atorvastatin plasma concentrations rapidly declined to subtherapeutic concentrations, and even 2 days after the end of ritonavir treatment, *C*_max_ was still 2.3-fold elevated when the original dose regimen was resumed (Fig. 5b). Simulations with the inter-individual variability in PK parameters highlight the pronounced variation in atorvastatin PK (Online Resource 9), whereas the ratio between baseline exposure and ritonavir-modulated exposure was stable (Online Resource 10).

### Safety and Tolerability

Trial medication and procedures were well tolerated. Adverse events were graded according to CTCAE 5.0. In total, three potentially related adverse events occurred in the atorvastatin group: Observed AEs were headache (*n* = 1; mild) and increased lipase (*n* = 2; mild and moderate), which resolved without treatment. Eight potentially related AEs were observed in the rosuvastatin group, two of them were moderate and six were mild. Reported AEs were headache (*n* = 5, 2 moderate), laboratory abnormalities (leucocytosis, increased neutrophils), and cough during ritonavir intake, and all resolved spontaneously.

## Discussion

In 2021, atorvastatin was the most commonly prescribed statin in Germany (over 160 million defined daily doses), while rosuvastatin saw the largest increase in prescriptions [36]. The widespread use of statins makes their combination with nirmatrelvir/ritonavir probable in clinical routine. Although ritonavir is known to alter the activity of numerous CYP isozymes and drug transporters, the effects and time-course of short-term, low-dose ritonavir treatment have not been studied in detail, and the expected DDIs and recommendations for outpatient management of potential DDIs are mostly based on studies with higher doses and longer treatment courses of ritonavir.

In our trial, the administration of 100 mg ritonavir twice daily for 5 days increased atorvastatin AUC_∞_ 4.76-fold, while both metabolites dropped below the LLOQ. This suggests at first glance inhibition of hepatic CYP3A4 metabolism. But surprisingly, there was no correlation between midazolam and atorvastatin ratios, and *t*_1/2_ did not change, which would have been expected if the increase in atorvastatin AUC was caused mainly by the inhibition of hepatic CYP3A4 metabolism. Ritonavir is also a potent inhibitor of drug transporters in vitro and in vivo and inhibits the efflux transporter p-gp [37] and the hepatic uptake transporters OATP1B1 and OATP1B3 [38], which mainly mediate hepatic atorvastatin uptake [39] but are not involved in midazolam disposition [40]. In addition, it has also previously been shown that the hepatic clearance of atorvastatin is mainly determined by the hepatic uptake via OATPs and not by (subsequent) CYP3A4 metabolism [41]. This indicates that effects of ritonavir causing irreversible intestinal inhibition of CYP3A4 [31], reversible inhibition of p-gp [42], and substantial blockage of hepatic atorvastatin uptake by OATP inhibition best explain the increase in atorvastatin exposure. The OATP effect is thought to be reversible as it disappeared after a wash-out of the drug in vitro [43].

In contrast, the AUC_∞_ of rosuvastatin was unchanged, and only *C*_max_ moderately increased, which is in line with previous findings [44, 45]. P-gp does not play a role in rosuvastatin uptake, and CYP metabolism is of limited relevance [46]. This situation may explain the clinically not relevant elevation of rosuvastatin AUC and the minor elevation of rosuvastatin *C*_max_. In heterozygous *SLCO1B1* carriers, rosuvastatin PK neither at baseline nor during ritonavir differed from carriers of two wild-type alleles, confirming earlier findings [47]. In contrast, rosuvastatin exposure in the homozygous SLCO1B1 *5/*15 carrier was 2.6-fold higher at baseline than observed in the other participants, consistent with the greater AUC of 19–68% reported in *SLCO1B1 521 T* > *C* carriers [48]**.** Interestingly, in this participant, ritonavir caused a 55% reduction of the AUC_∞_. Such a paradoxical reaction has already been reported with a different OATP1B1 inhibitor in the same individual [49]. The mechanism of such a phenomenon is not clear but might indicate the activation of the (defective) transporter by ritonavir. Indeed, the activation of OATP1B transporters by drugs that are not transported has already been demonstrated [50], but to our best knowledge, this has not been investigated for ritonavir.

Serious adverse effects of statins, such as rhabdomyolysis, can occur after only 1 week of high statin exposures [19, 20]. The almost 5-fold increase in exposure to atorvastatin could therefore pose a risk to patients, especially if they take high-maintenance doses or other risk factors are present. On the other hand, acute discontinuation of statins may pose vascular risks, not only in patients with acute vascular syndromes [51, 52] but also in patients with chronically very high cardiovascular risk [53] and in patients with COVID-19 [54]. Temporary discontinuation of statins in patients with COVID-19, as sometimes recommended [3], was shown to be detrimental as discontinuation of statins can lead to higher in-hospital mortality [54]. Based on the observed PK changes, we therefore simulated how atorvastatin treatment schedules could be changed to keep atorvastatin exposure uninterrupted and within safe and effective margins.

Using PK modelling of atorvastatin plasma concentrations, we explored various alternative treatment regimens during ritonavir treatment that are suitable to avoid excessive atorvastatin exposure and associated adverse effects. According to this modelling, abrupt atorvastatin discontinuation rapidly leads to subtherapeutic plasma concentrations, and if atorvastatin is restarted even after a 2-day pause, plasma concentrations are still expected to temporarily double. The latter finding is presumably explained by irreversible CYP3A4 inhibition, which only gradually recovers after the discontinuation of ritonavir [31]. For patients on high potentially dangerous maintenance doses, the best dosing scheme was a step-wise dose reduction followed by an incremental increase to the original atorvastatin dose as follows: administration of the full dose on the first nirmatrelvir/ritonavir treatment day (at the same time as ritonavir), a half of the atorvastatin dose on day 2, and a quarter of the dose on days 3–6. After the discontinuation of nirmatrelvir/ritonavir, the administration of half the dose until day 9 and restart of the original dose from 5 days after discontinuation of nirmatrelvir/ritonavir. The second-best alternative to minimize exposure changes is to reduce the patient’s atorvastatin dose to either half for patients receiving 20 mg or to a quarter for patients receiving 40 mg or higher doses until 2 days after the end of nirmatrelvir/ritonavir treatment and then continue with the original dose. Some of these regimens require that the tablets be divided into quarters, which is unfortunately often not the case [29]. For patients with 10 mg atorvastatin, maintaining the original dose is usually the preferred option as exposure will likely not exceed exposures observed with the maximum licensed dose.

## Limitations

This trial analyzed the change in the exposure of single low doses of atorvastatin or rosuvastatin on the fifth day of ritonavir treatment compared to baseline. While this design assessed maximum inhibition, it cannot inform about the time course of inhibition in the first days of treatment. However, thorough analyses with the CYP3A4 marker substrate midazolam revealed that profound inhibition already occurs after the first ritonavir dose [23]. Furthermore, because the PK of atorvastatin is slightly non-linear [55], the effect of ritonavir on higher doses of atorvastatin may differ slightly from these estimates. In addition, we used a mechanism-based model that plausibly explains prolonged ritonavir effects by irreversible CYP3A inhibition. However, possible additional reversible ritonavir effects on drug transporters could not be distinguished by our trial design.

## Conclusion

In conclusion, our trial showed an almost 5-fold increase in atorvastatin exposure in healthy volunteers while rosuvastatin exposure was only mildly altered. Semi-mechanistic PK modelling based on enzyme turnover, to reflect irreversible inhibition, suggests that boosting doses of ritonavir achieve nearly maximum inhibition after 24–48 h. Therefore, temporary dose reductions of atorvastatin are recommended for patients maintained on daily doses exceeding 10 mg. The results of this trial suggest that patients receiving rosuvastatin do not need to change their dose during short courses of ritonavir.

## Supplementary Information

Below is the link to the electronic supplementary material.

Supplementary file1 (PDF 695 KB)

## Data Availability

Source data and the dataset generated during the trial are available from the corresponding author upon reasonable request.
