# Supplementary material for: Influence of a Short Course of Ritonavir Used as Booster in Antiviral Therapies Against SARS-CoV-2 on the Exposure of Atorvastatin and Rosuvastatin
Source: Cardiovasc Drugs Ther. 2023 Dec 19;39(2):325–35. doi: 10.1007/s10557-023-07538-w (PMC11954719; doi:10.1007/s10557-023-07538-w)
Supplement: Supplementary file 1 — Supplementary file1 (PDF 695 KB) [file 10557_2023_7538_MOESM1_ESM.pdf]

Supplementary Material

Influence of a short course of ritonavir as used as booster in antiviral therapies against SARS-CoV-2 on the exposure of atorvastatin and rosuvastatin

Authors and affiliations

Evelyn Krohmer, Brit Silja Rohr, Felicitas Stoll, Katja S. Gümüs, Mariano Bergamino, Gerd Mikus, Max Sauter, Jürgen Burhenne, Johanna Weiss, Andreas D. Meid, David Czock, Antje Blank, Walter E. Haefeli

Department of Clinical Pharmacology and Pharmacoepidemiology, Heidelberg University Hospital, Im Neuenheimer Feld 410, 69120 Heidelberg, Germany

Corresponding author:

Prof. Walter E. Haefeli, MD, FBPhS

Email: walter.emil.haefeli@med.uni-heidelberg.de

Online Resource Table 1 Atorvastatin population pharmacokinetic parameters from the semi-mechanistic model.

Parameter	Estimate	Unit	Inter-individual variability (CV %)
V_c/F	2507	[L]	89.5
CL/F	540	[L/h]	79.4
V_t/F	4040	[L]	-
CL_d/F	2387	[L/h]	-
F	1[a]		59.9
t_{abs}	1.20[b]	[h]	-
k_{deg}	0.00866[a]	[1/h]	-
RTV_{effect}	1.00[b]		-
$RTV_{effect,gut}$	0.57		-
$RTV_{effect,tabs}$	1.71[b]		-
Proportional error	0.50		

CL/F: apparent clearance after oral administration; CL_d/F: apparent distribution clearance; CV: coefficient of variation; F: bioavailability; k_{deg}: CYP enzyme degradation-rate constant; RTV_{effect}: ritonavir-induced relative change of CYP activity after a ritonavir dose as used to modify drug elimination; $RTV_{effect,gut}$: ritonavir-induced relative change of CYP activity after a ritonavir dose used to modify bioavailability; $RTV_{effect,tabs}$: ritonavir-induced relative change of zero-order absorption time; t_{abs}: zero-order absorption time; V_c/F: apparent volume of the central compartment; V_t/F: apparent volume of peripheral compartment; [a]: Value fixed according to clinical reasoning or literature results. [b]: Value fixed according to separate analyses.

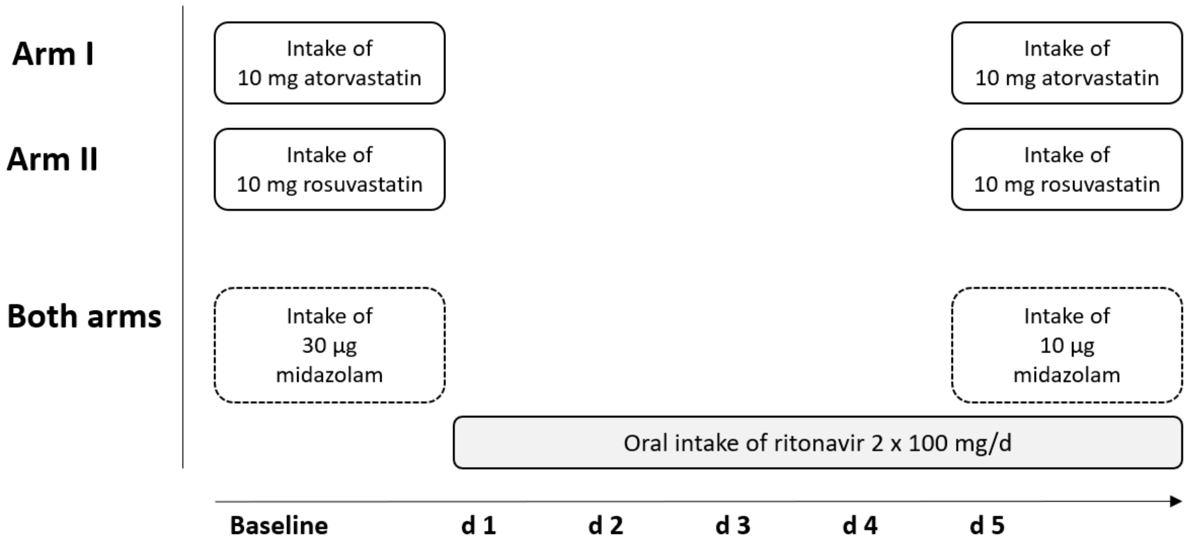

Online Resource 1: Trial design to evaluate the perpetrator effects of a 5-d ritonavir treatment regimen (2 x 100 mg/d) on the single-dose pharmacokinetics of 10 mg atorvastatin or 10 mg rosuvastatin and a microdose of midazolam in eight healthy volunteers per study arm.

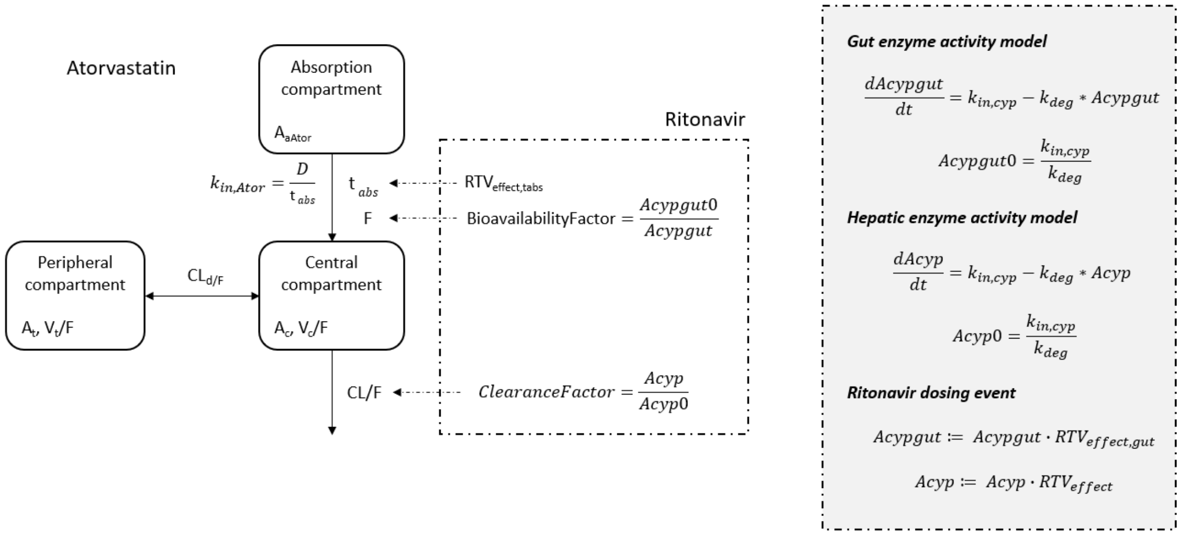

Online Resource 2: Final atorvastatin pharmacokinetic model. AaAtor: amount in absorption compartment; A_c and A_t: amount in central and peripheral compartments; Acyp0 and Acypgut0: relative activity of hepatic and intestinal CYP-enzyme before intervention (set at 1); Acyp and Acypgut: relative activity of hepatic and intestinal CYP-enzyme (initial values set at 1); C_c: plasma concentration; CL_d/F, apparent distribution clearance; CL/F, apparent clearance after oral administration; k_{deg}: 1st-order degradation rate constant; $k_{in,Ator}$: zero-order absorption rate constant; $k_{in,cyp}$: hepatic/ intestinal CYP synthesis rate; RTV_{effect}: ritonavir-induced relative change of CYP activity after a ritonavir dose as used to modify drug elimination; $RTV_{effect,gut}$: ritonavir-induced relative change of CYP activity after a ritonavir dose used to modify bioavailability; $RTV_{effect,tabs}$: ritonavir-induced relative change of zero-order absorption time; t_{abs}: zero-order absorption time; V_c/F: apparent volume of the central compartment V_t/F: apparent volume of peripheral compartment.

Online Resource 3: Code of the pharmacokinetic atorvastatin model fitted with the nlmixr2 package and simulated with the rxode2 package (second part). Apparent zero-order absorption time (and its modulation by ritonavir) were considered as fixed values in the data frame and event tables, respectively.

```
nlmixr2_model <- function() {
# parameter definition and inital estimates (starting values)
  ini({
   # central volumen of distribution (fixed to baseline estimate)
     tVc <- fix(log(exp(fit_alone$fixef['tVc']))); label("Vc")
   # (systemic) clearance (fixed to baseline estimate)
     tCl <- fix(log(exp(fit_alone$fixef['tCl']))); label("Cl")
   # peripheral volumen of distribution (fixed to baseline estimate)
     tVt <- fix(log(exp(fit_alone$fixef['tVt']))); label("Vt")
   # inter-compartmental clearance (fixed to baseline estimate)
     tCld <- fix(log(exp(fit_alone$fixef['tCld']))); label("Cld")
   # bioavailability (fixed to 1)
     tf <- fix(log(1)); label("F")
   # RTV effect on (remaining) systemic clearance (fixed to Null effect, no further influence
detectable)
     tRTVeffect <- fix(log(1)); label("RTVeffect")
   # RTV effect on first-pass (gastro-intestional clearance)
     tRTVeffectgut <- c(log(0.5), log(0.8), log(0.9)); label("RTVeffectgut")
   # Inter-individual variability (IIV)
     eta.f ~ 0.3
     eta.Vc ~ 0.6
     eta.Cl ~ 0.5
   # proportional error model
     prop.err <- 0.4
  })
# model block with the error specification and model specification
  model({
   # model-defined initial values of state variables
     Acyp(0)<-1;
     Acypgut(0)<-1;

   # Parameters and IIV
     Vc <- exp(tVc + eta.Vc)
     Cl <- exp(tCl + eta.Cl)
     Vt <- exp(tVt)
     Cld <- exp(tCld)
     f <- exp(tf + eta.f)
     RTVeffect <- exp(tRTVeffect)
     RTVeffectgut <- exp(tRTVeffectgut)

   # parameter calculations
     kdeg <- 0.00866;
     R0=kdeg;

   # output as concentration
     Cc = Ac / Vc;
     Ct = At / Vt;

   # ATV Pharmakokinetic model
     # dosing compartment (used for zero-order absorption)
        d/dt(AaAtor) = 0
     # central compartment
        d/dt(Ac) = - ((Cl *(Acyp))*Cc) + (AaAtor * f *(1/Acypgut)) - (Cld * Cc) + (Cld * Ct)
     # peripheral compartment
        d/dt(At) = (Cld * (Cc - Ct))

   # CYP activity model: (time-dependent) covariate influence of RTV administration
     d/dt(RTV_adminis) = 0;
     d/dt(Acyp) = (R0-(kdeg*(Acyp))) - (Acyp - (Acyp*RTVeffect^(RTV_adminis)));
     d/dt(Acypgut) = (R0-(kdeg*(Acypgut))) - (Acypgut - (Acypgut*RTVeffectgut^(RTV_adminis)));

   # output (dependent) variable
     ATV <- Cc * 1000;
     ATV ~ prop(prop.err)
  })
}
```

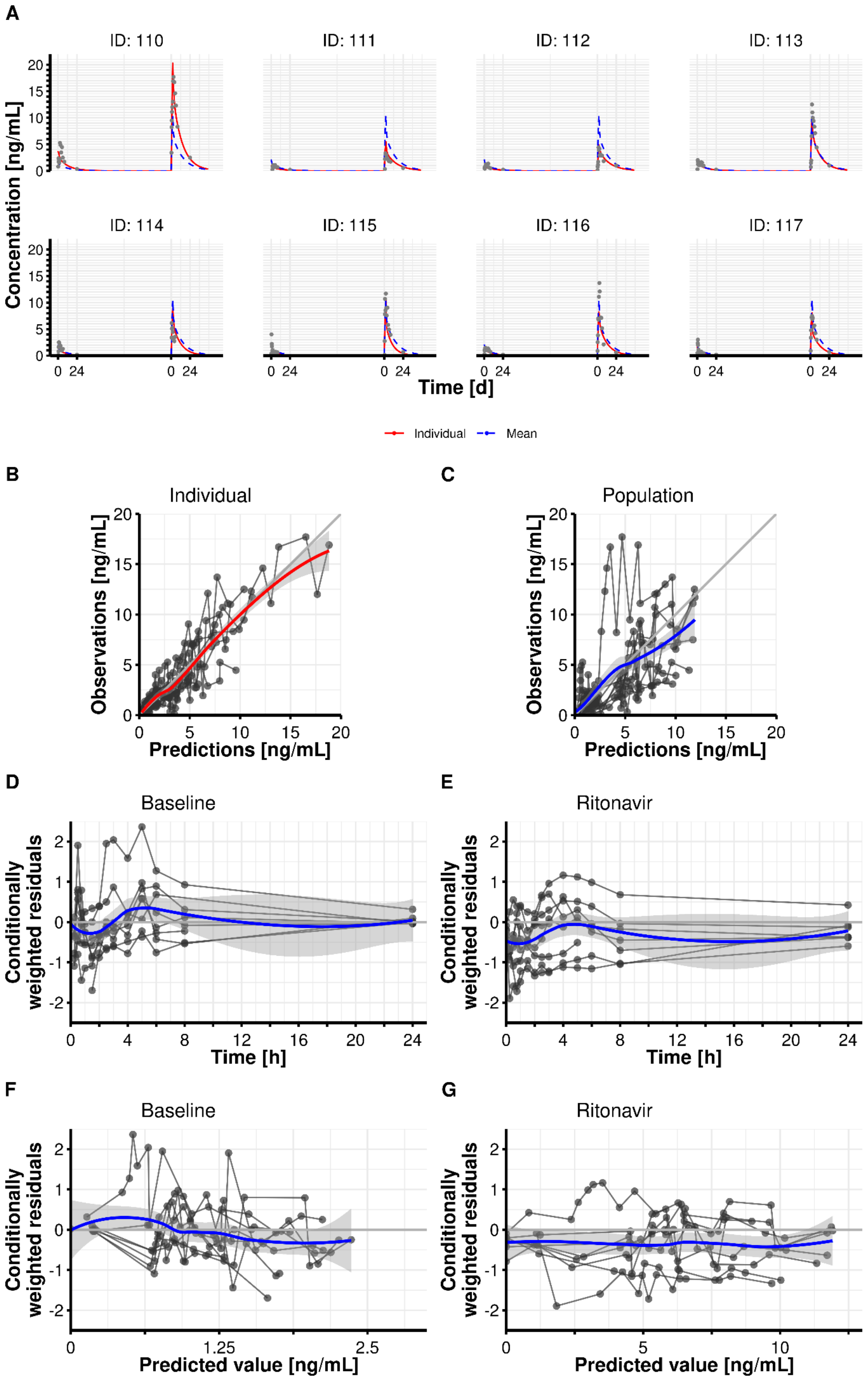

Online Resource 4: Model diagnostics for (visual) inspection of goodness of fit for the final pharmacokinetic model of atorvastatin concentrations (A: Individual and population fits; B/C: Observed versus predicted values of individual and population fits; D/E: Residuals versus time during baseline and under ritonavir treatment; F/G: Residuals versus predicted values during baseline and under ritonavir treatment).

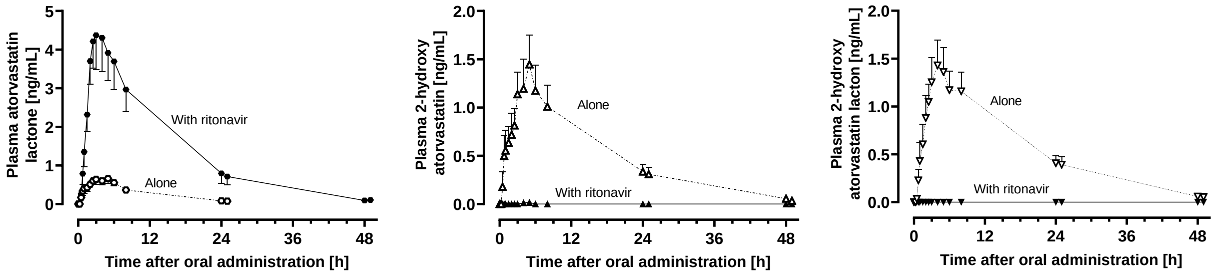

Online Resource 5: Mean (± SEM) plasma concentration-time profiles of atorvastatin lactone (left), 2-hydroxy atorvastatin acid (middle) and 2-hydroxy atorvastatin lactone (right) before and on the fifth day of ritonavir administration (2 x 100 mg/d) in eight healthy volunteers.

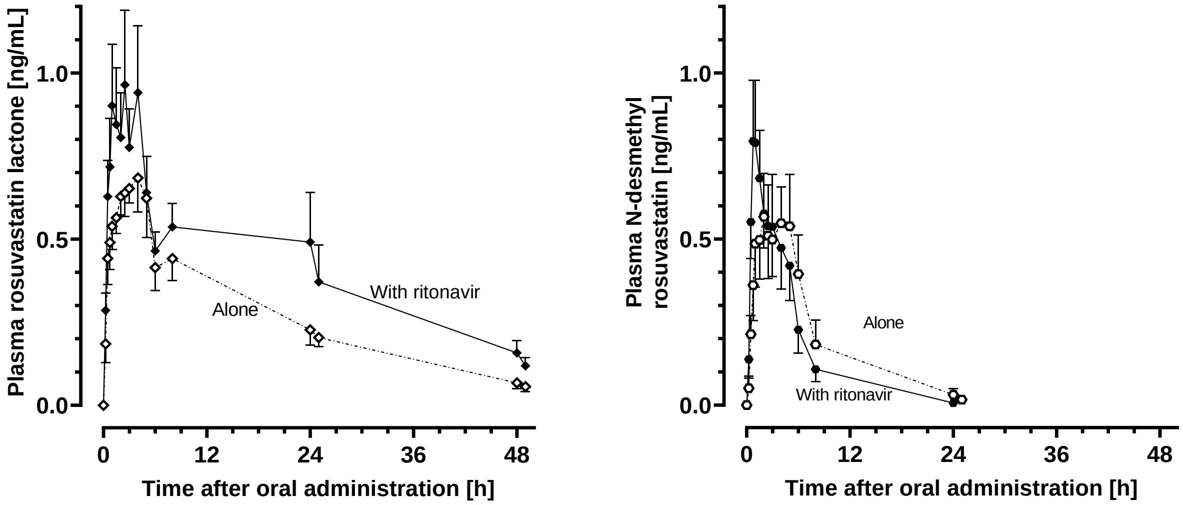

Online Resource 6: Mean (± SEM) plasma concentration-time profiles of rosuvastatin lactone (left) and N-desmethyl rosuvastatin (right) before and on the fifth day of ritonavir administration (2 x 100 mg/d) in eight healthy volunteers.

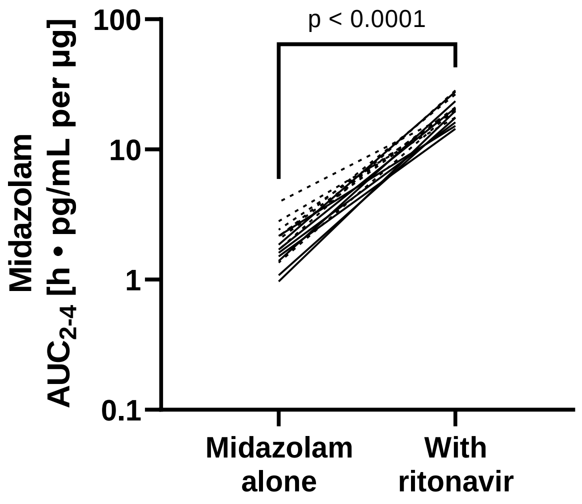

Online Resource 7: Change in midazolam AUC_{2-4} before and on the fifth day of ritonavir (2 x 100 mg/d) in 16 healthy volunteers. Solid lines show participants of the atorvastatin group and dashed lines show participants of the rosuvastatin group. AUC_{2-4}: area under the concentration-time curve from 2-4 h normalized to 1 µg for the two different doses administered.

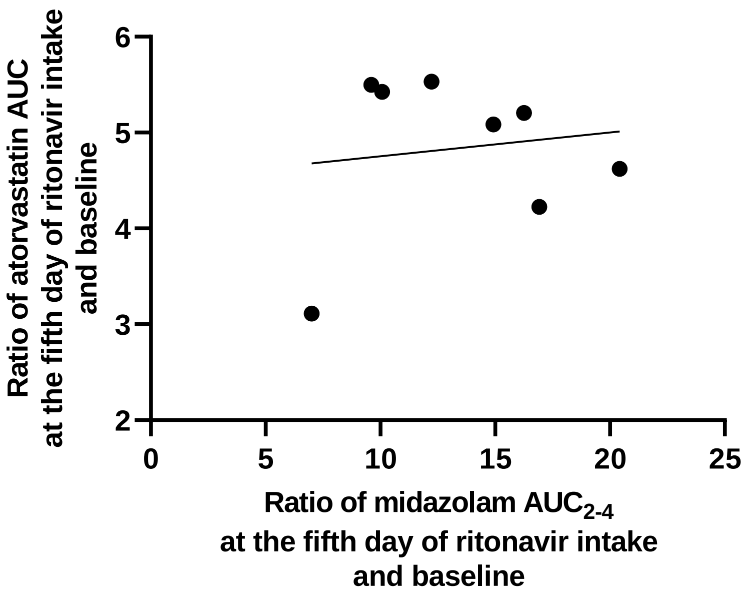

Online Resource 8: Regression of individual midazolam AUC ratios of the increase at the fifth day of ritonavir intake ($r^2 = 0.02$. $p = 0.75$) as a marker mainly influenced by CYP3A4 activity with the ratio of the atorvastatin AUC increase.

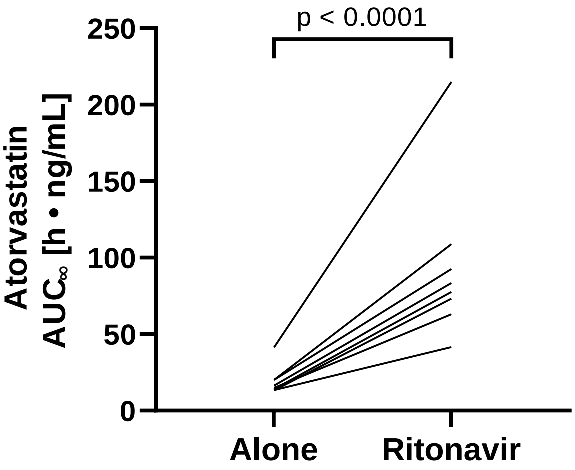

Online Resource 9: Impact of 5 d of ritonavir (2 x 100 mg/d) on the exposure of atorvastatin after administration of a single dose of 10 mg to eight healthy volunteers. AUC_∞: area under the concentration-time curve extrapolated to infinity.

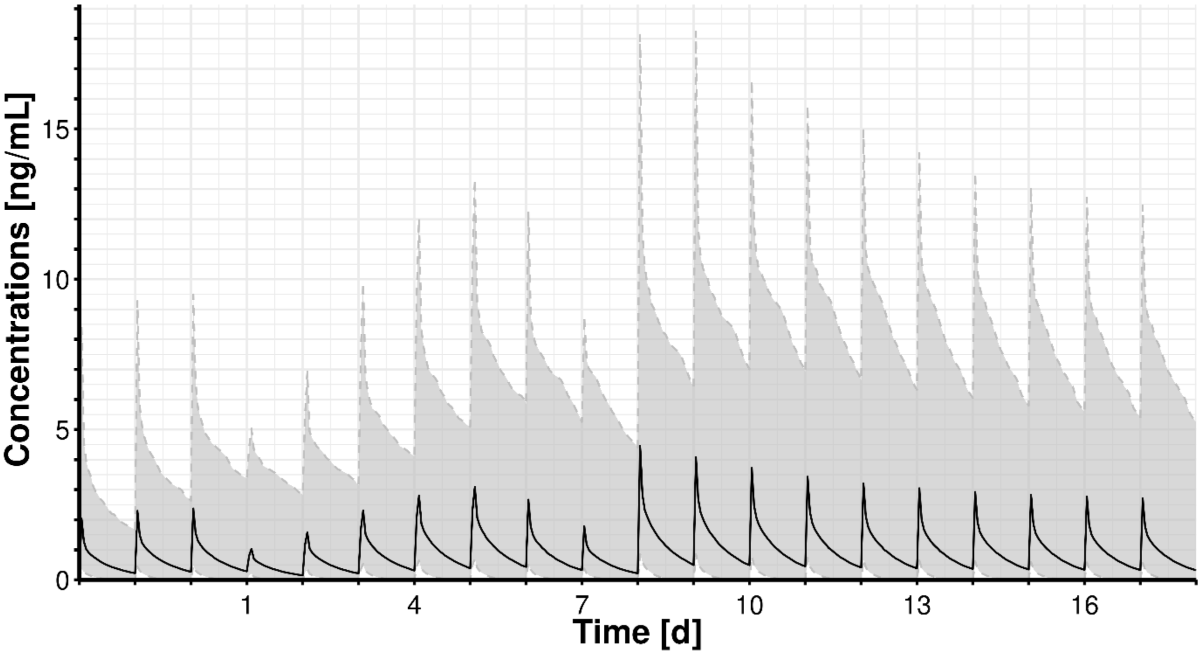

Online Resource 10 Simulation with inter-individual variability in 1000 virtual patients most practical dosing regimen (e) according to Figure 5. The solid line marks the median trajectory, while 95 % of all predicted values fall within the grey-shaded region (dashed lines indicate upper and lower bounds).
